# Effects of Landscape Pattern Change on Water Yield and Nonpoint Source Pollution in the Hun-Taizi River Watershed, China

**DOI:** 10.3390/ijerph17093060

**Published:** 2020-04-28

**Authors:** Min Zong, Yuanman Hu, Miao Liu, Chunlin Li, Cong Wang, Xiaoying Ping

**Affiliations:** 1 CAS Key Laboratory of Forest Ecology and Management, Institute of Applied Ecology, Chinese Academy of Sciences, Shenyang 110016, China; 2 College of Resources and Environment , University of Chinese Academy of Sciences, Beijing 100049, China

**Keywords:** WYLD, NPS, landscape pattern, SWAT

## Abstract

Understanding the influence of landscape pattern changes on water yield (WYLD) and nutrient yield is a key topic for water resource management and nonpoint source (NPS) pollution reduction. The annual WYLD and NPS pollution were estimated in 2004 and 2015 with the calibrated and validated Soil and Water Assessment Tool (SWAT) in the Hun-Taizi River watershed. The impact of land use and landscape pattern changes on the annual WYLD and NPS loading changes were analyzed with a boosted regression tree (BRT) and redundancy analysis (RDA). The results showed that WYLD had a positive correlation with dry farmland and built-up area; however, a negative correlation with paddy field and water area, with the relative contribution of 42.03%, 23.79%, 17.06%, and 13.55%, respectively. The change in nutrient yield was positively correlated with changes in dry farmland, built-up area, and water area but negatively with forestland, according to the BRT model. Landscape patterns had an important influence on WYLD and NPS pollution. A large unfragmented forestland may improve water quality, while a large concentrated dry farmland results in water quality deterioration due to NPS pollution. Water quality is more likely degraded when land uses are complex and scattered with many small patches in a forestland dominated watershed.

## 1. Introduction

Water yield (WYLD) is of great importance as it supplies water resources to human beings and natural resources [1]. Under the background of rapid urbanization and economic development, water shortages and water degradation have become increasingly severe in many watersheds in China [2,3]. Point source (PS) pollution and nonpoint source (NPS) pollution have been identified as key triggers of deteriorating water quality [4]. PS pollution has been fairly controlled, while water quality has not significantly improved, largely due to NPS pollution with a wide range of pollution sources and complex uncertainties [5,6]. Total nitrogen (TN) and total phosphorus (TP) are the major pollutants resulting from agricultural and urban NPS and have become a major contributor to water-related problems, such as river contamination, aquatic ecosystem deterioration, and severe eutrophication [7].

The relationship between NPS pollution and landscape pattern changes has substantial implications for TN and TP yield control. Field monitoring and model prediction are the two main methods used to calculate NPS. However, field monitoring is expensive, time-consuming, and regionally characterized, limiting the development of the method. With the development of geographic information systems (GIS), remote sensing (RS), and computer technology, many hydrological models have been developed to simulate hydrological processes and water quality. Many physically distributed models have been successfully developed for NPS simulation, such as annualized agricultural nonpoint source [8]; areal nonpoint source watershed environment response simulation [9]; better assessment science integrating point and nonpoint sources [10]; chemicals, runoff, and erosion from agricultural management systems [11]; hydrological simulation program fortran [12]; and Soil and Water Assessment Tool (SWAT) [13]. The SWAT model has been used worldwide because of its high level of prediction accuracy [14,15,16].

The SWAT model is widely used to simulate streamflow, sediment yield, and NPS loading effectively, which are greatly affected by changes in rainfall, land use, and landscape pattern during a short period [17]. With urbanization and agricultural activities, land use and landscape pattern are strongly related to human activities and government policies, especially in China. Studies have found that land use changes dramatically affect hydrological processes [18] and NPS loading [19]. However, WYLD and NPS have highly nonlinear responses to land use changes [1,20], resulting in different results in previous studies. Landscape configurations are sensitive predictors of water quality [21]. Changes in the landscape configuration in a watershed result in changes in the flows of energy and nutrients, including the flows of solar radiation, temperature, evapotranspiration, surface runoff, discharge, nutrient transfer, soil erosion, and sediments [22]. A comprehensive research considering the influence of land use and landscape pattern changes on WYLD and water quality is necessary.

In recent years, urban sprawl has accelerated in the Hun-Taizi River watershed due to the policy of “Revitalization of Old Industrial Bases in Northeast China” proposed in 2003. The policy has caused considerable land use and landscape pattern changes [23]. With the control of PS pollution, intense urbanization and agricultural activities have led to the continuous increase in regional NPS pollution loads. NPS pollution is not only a serious threat to the security of drinking water and agricultural irrigation but also to the sustainable economic and social development of the Hun-Taizi River watershed. The objectives of this study are (1) to evaluate the WYLD and NPS loading in 2004 and 2015 of the Hun-Taizi River watershed; (2) to quantify the contribution of land use changes to WYLD and NPS pollution using a boosted regression tree (BRT) model; and (3) to examine the relationship between landscape pattern changes and WYLD and NPS loading using the redundancy analysis (RDA) method.

## 2. Materials and methods

### 2.1. Study Area

**Figure 1:**
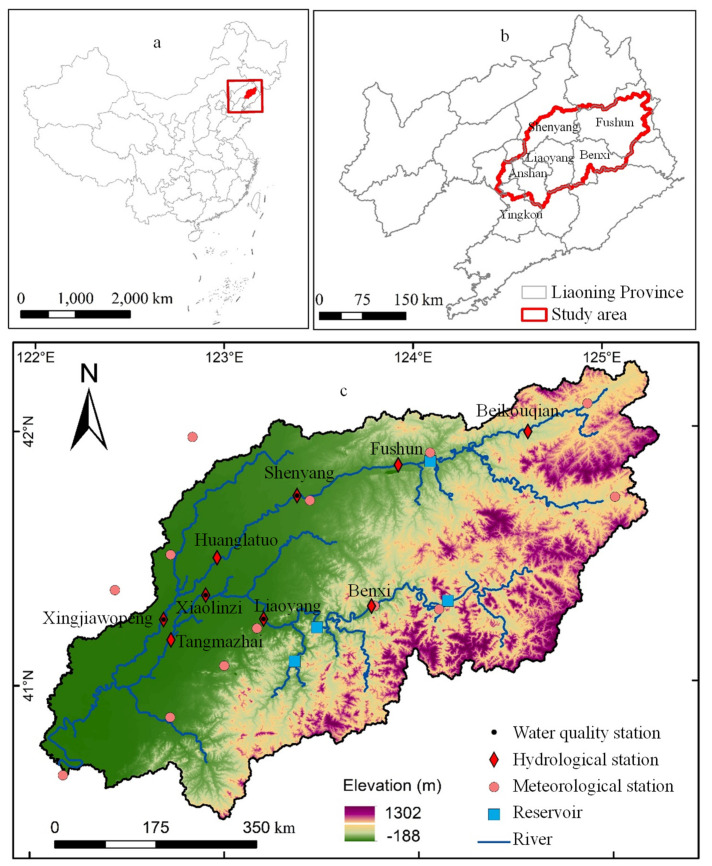
Location of the study area. (**a**) China; (**b**) The study area in Liaoning Province; (**c**) The study area.

The Hun-Taizi River watershed (121° 57′–125° 20′ E, 40° 27′–42° 19′ N), in the central part of Liaoning Province, northeastern China (Figure 1), covering an area of 2.73 × 10^4^ km^2^, was selected as the study area. The Hun-Taizi River watershed is a subbasin of the Liao River and consists of the Hun River (415 km in length, a drainage area of 11,481 km^2^), the Taizi River (413 km in length, a drainage area of 13,883 km^2^), and the Daliao River (96 km in length) that flows into the Bohai Sea through Yingkou city. The study area has a warm temperate semihumid monsoon climate with an average annual temperature of 8.7 °C, and the mean maximum and minimum temperatures were 28.9 and −15.7 °C in July and January, respectively, from 1980–2016. The average annual precipitation of the last 30 years in the watershed was approximately 700 mm (ranging from 610–780 mm), although 70% of the precipitation falls in the monsoon season (June to September). The intra-annual distribution of streamflow is similar to that of precipitation, with more than 70% occurring from June to September. There are four main reservoirs (Dahuofang, Guanyinge, Shenwo, and Tang), which are the most important sources of drinking water and irrigation water for Liaoning Province. The study area covers most of the central Liaoning Urban Agglomeration, which is one of ten urban agglomerations in China, and includes the cities of Shenyang, Anshan, Fushun, Benxi, Liaoyang, and Yingkou. The study area had a residential population of 1.86 × 10^7^ and produced 47.03% of the total gross domestic product (GDP) of Liaoning Province in 2016 [24].

### 2.2. SWAT Model

#### 2.2.1. SWAT Model Description

The SWAT model (United States Department of Agriculture (USDA), Texas, USA) is a physically based, long-term, continuous, semidistributed watershed-scale hydrological model developed by the Agriculture Research Service (ARS) of the United States Department of Agriculture (USDA) operating on daily or monthly time steps [25]. The SWAT model was designed to assess the impact of land management practices on hydrology and water quality in large complex watersheds with varying soils, land uses, and management conditions over long time periods [26]. In this study, the 2012 SWAT model was used to determine the land use change impacts on NPS pollution in the Hun-Taizi River watershed. Detailed descriptions of the SWAT model can be found in the literature [15,25,27].

In this study, the modified soil conservation services curve number (SCS-CN, U.S. Department of Agriculture, Bronx, NY, USA) was used to estimate surface runoff from daily precipitation, and a modified rational method was used to quantify peak runoff rates. The SWAT model simulates nutrient transport and transformation with the QUAL2E model at each hydrologic response unit (HRU). The Penman–Monteith method was used to calculate potential evapotranspiration (PET), the Muskingum method was used to route channel flow, and Manning’s formula was used to determine the watershed concentration time.

#### 2.2.2. Data Collection and Model Parameter Setting

**Figure 2:**
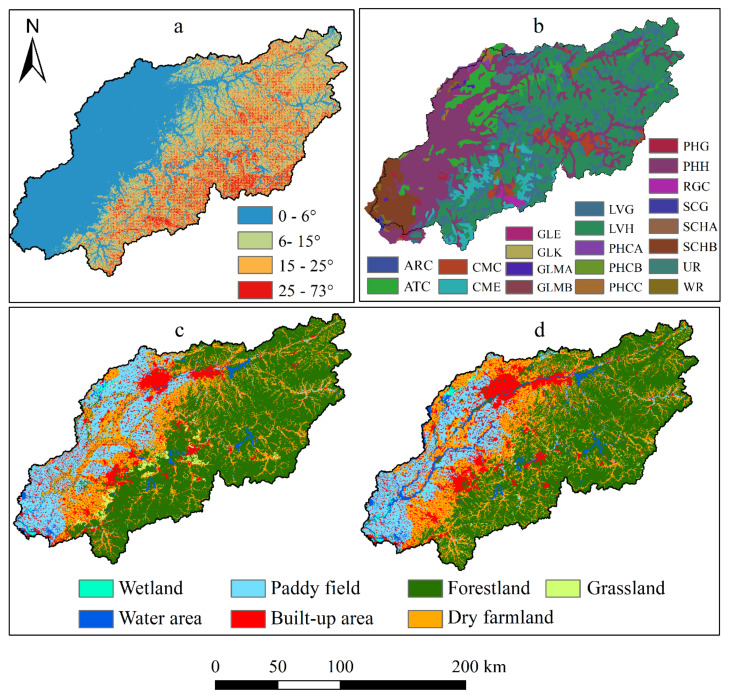
The data required for the Soil and Water Assessment Tool (SWAT) model. (**a**) Slope; (**b**) soil types; (**c**) 2004 land use; (**d**) 2015 land use. Calcaric Arenosols (ARC), Cumulic Anthrosols (ATC), Calcaric Cambisols (CMC), Eutric Cambisols (CME), Eutric Gleysols (GLE), Calcic Gleysols (GLK), Mollic Gleysols (GLMA and GLMB), Gleyic Luvisols (LVG), Haplic Luvisols (LVH), Calcaric Phaeozems (PHCA, PHCB, and PHCC), Gleyic Phaeozems (PHG), Haplic Phaeozems (PHH), Calcaric Regosols (RGC), Gleyic Solonchaks (SCG), Haplic Solonchaks (SCHA and SCHB), Urban, mining, etc. (UR), Water bodies (WR).

The database for the SWAT model is presented in Table 1 and Figure 2. The geographic data (topographic data, land use, and soil map) used were a grid format with a resolution of 30 × 30 m. Using the “Feature to Raster tool” makes the digital elevation model (DEM) to grid format, and the “Resample tool” changes the soil map to resolution in ArcGIS 9.1 (ESRI, RedLands, CA, USA). Land use was interpreted with Landsat images, including paddy field, dry farmland, forestland, grassland, water area, built-up area, and wetland. The accuracies of interpretation were 81.3% and 87.6% compared with 270 field survey points. Meteorological data during 2001–2016 was collected from 13 meteorological stations in the study area. Monthly observed streamflow data were collected from nine hydrological stations from 2001 to 2014. The monthly maximum and minimum discharge data of the four reservoirs were collected during 2001–2014. Due to limits on the monitoring data of water quality, only four stations were acquired for the period of 2001–2005. PS emissions and NPS pollution were evaluated by the export coefficient method [28].

In this study, the Hun-Taizi River watershed was divided into 55 subbasins, with the threshold area being set at 35,000 ha with a total area of 2,774,045 ha. The slope of the land was grouped into four grades (≤ 6°, 6–15°, 15–25°, ≥25°). The land uses, soil map, and land slopes overlapped with each other with thresholds of 2% each to define the HRUs. In total, 1846 HRUs were defined with uniform parameters and variables being used in each unit.

#### 2.2.3. Model Calibration and Validation

Before the SWAT model is applied, it has to be properly calibrated and validated for ascertaining the reliability and accuracy of the model. An automatic parameter estimation procedure SWAT-calibration and uncertainty program (SWAT-CUP, version 2012, EAWAG, Neprash Cooperation, and Texas A&M University, TX, USA), was used to calibrate and validate the model [15]. Considering the large uncertainties related to the watersheds and the high efficiency and accuracy of the calibration, the sequential uncertainty fitting version-2 (SUFI-2) method was used in this study [29]. Initially, a sensitivity analysis was required in the first step before the calibration was performed. The model was run using a two-year warm-up period from 2001 to 2002. The SWAT was first calibrated and validated for streamflow from 2003 to 2010 and 2011 to 2014, respectively. Calibration and validation of TN and TP were performed for the periods 2003–2004 and 2005, respectively.

The R^2^ and the Nash-Sutcliffe efficiency coefficient (E_NS_) [30] have been widely used as two criteria for evaluating a model’s performance in calibration and validation. Generally, the SWAT model performance of calibration/validation criteria is as follows: R^2^ and E_NS_ greater than 0.9, 0.75–0.9, 0.5–0.75, 0.25–0.5, 0–0.25, and below 0 correspond to model performances of “excellent”, “very good”, “good”, “fair”, “poor”, and “unsatisfactory”, respectively [31,32,33].

#### 2.2.4. Model Application

**Figure 3:**
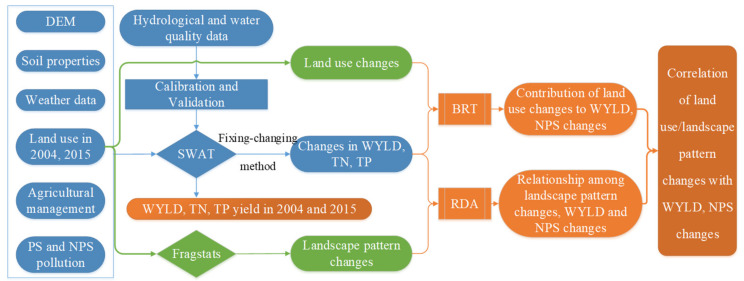
Schematic framework of the study. Digital elevation model (DEM), Point source (PS), Nonpoint source (NPS), Soil and Water Assessment Tool (SWAT), Water yield (WYLD), Total nitrogen (TN), Total phosphorus (TP), Bboosted regression tree (BRT), Redundancy analysis (RDA).

A “fixing-changing” method [34,35] was used to detect the effect of landscape pattern changes on WYLD and nutrient yield changes. The calibrated model was run for each land use map (2004 and 2015), maintaining the DEM and soil data constant from January 2001 to December 2016. The simulated results were used to quantify the impacts of land use and landscape pattern changes on streamflow and nutrient yield changes (Figure 3).

### 2.3. Statistical Analysis

#### 2.3.1. Boosted Regression Tree

Boosted regression tree (BRT) was chosen to analyze effect factors of WYLD and nutrient yield, which is a flexible regression modeling technique that can accommodate different types of variables and missing data, and is immune to the influence of extreme outliers and the inclusion of irrelevant variables, can fit complex nonlinear relationships, and automatically handle interaction effects between predictors [36]. The BRT estimates the contribution of each predictor through a hierarchical binary splitting procedure that maximizes differences among variables. At each step, a forward stagewise process reserves existing trees that were already built and adds new trees by reweighing residuals from previous trees. Therefore, the BRT generates thousands of trees and a mean relative contribution [1]. Detailed descriptions of the BRT model can be found in the literature [1,37,38].

The changes in WYLD and nutrient yield across two simulations was developed by using land use maps for 2004 and 2015, as the dependent variables. The independent variables were the six land use types changes (dry farmland, paddy field, forestland, grassland, water area, and built-up area). For this study, three BRT models were constructed to analyze the relative contribution of changes in individual land use to WYLD, TN, and TP yields. BRT models are developed in the R software (version 3.6.1), specifically by using its package “gbm”, with “gbm” functions. The BRT models used in this study have a learning rate of 0.005, a bag fraction of 0.5, a tree complexity of 5, and a 10-fold cross validation [36]. In addition, figures were drawn with the package “ggplot2” in R.

#### 2.3.2. Landscape Metrics

Landscape metrics are usually used to describe landscape spatial patterns [39]. According to landscape metrics meaning studies [40,41], the percentage of landscape (PLAND), largest patch index (LPI), patch density (PD), edge density (ED), landscape shape index (LSI), perimeter area fractal dimension (PAFRAC), interspersion juxtaposition index (IJI), aggregation index (AI), contagion index (CONTAG), Shannon’s diversity index (SHDI), and land use distance to river were selected as landscape pattern metrics. Land use distance to rivers quantitatively reflects the distribution patterns of land use types relative to rivers [19], calculating the average distance from the centroid of each land use type to the nearest river. PLAND, LPI, PD, ED, LSI, PAFRAC, IJI, and AI were calculated at the class level. LPI, PD, ED, LSI, PAFRAC, IJI, AI, CONTAG, SHDI, and land use distance to river were calculated at the landscape level. The landscape metrics were calculated using FRAGSTATS 3.3 (University of Massachusetts in Amherst, Amherst, MA, USA).

#### 2.3.3. Redundancy Analysis

The redundancy analysis (RDA) approach was used to analyze the relationship among landscape pattern changes and WYLD as well as nutrient yield changes in each subbasin. A detrended correspondence analysis (DCA) was first performed to select the most suitable ordination method. As the gradient length was less than 3 (1.79), the RDA was selected. Then, the major factors that affected the dependent variables were chosen according to the contribution and significance values by using the interactive forward-selection method. Finally, the RDA was conducted based on the selected environmental factors. The analysis was undertaken using the universal vegetation quantitative analysis software CANOCO 4.5 [39,42]. Triplots were drawn to evaluate the correlation of landscape metrics with WYLD and NPS loads.

## 3. Results

### 3.1. SWAT Model Calibration and Validation

**Figure 4:**
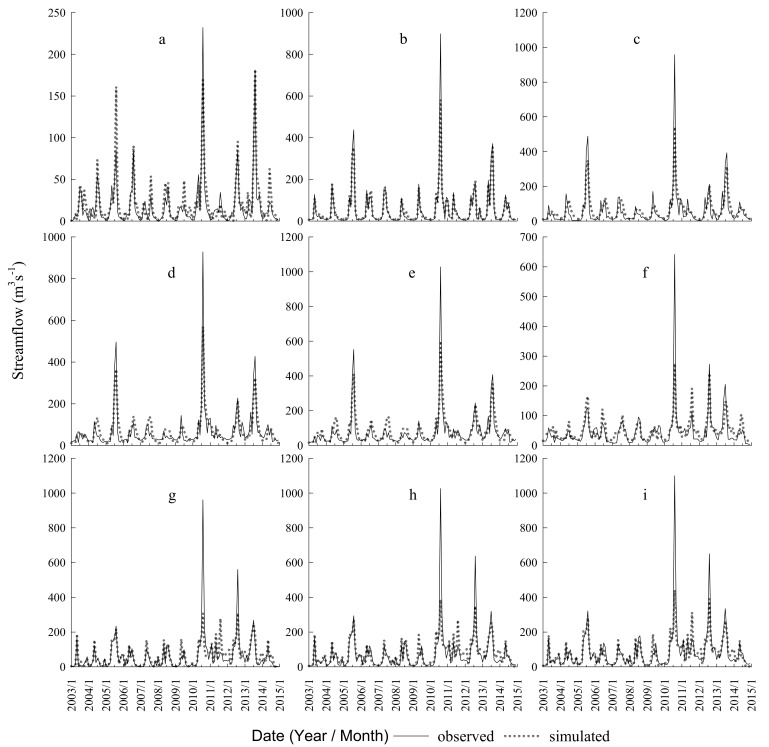
Comparison between the observed and simulated monthly streamflow values at nine stations for the calibration period (2001–2010) and validation period (2011–2014). (**a**) Beikouqian station; (**b**) Fushun station; (**c**) Shenyang station; (**d**) Huanglatuo station; (**e**) Xingjiawopeng station; (**f**) Benxi station; (**g**) Liaoyang station; (**h**) Xiaolinzi station; and (**i**) Tangmazhai station.

**Figure 5:**
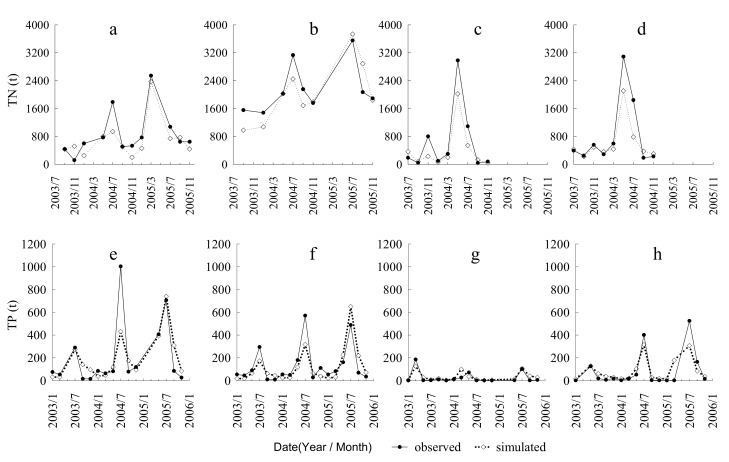
Comparison between the observed and simulated monthly nutrient yield at four stations for the calibration period (2003–2004) and validation period (2005). (**a**) Shenyang station; (**b**) Xingjiawopeng station; (**c**) Liaoyang station; (**d**) Xiaolinzi station; (**e**) Shenyang station; (**f**) Xingjiawopeng station; (**g**) Liaoyang station; (**h**) Xiaolinzi station.

The observed monthly streamflow data and odd-numbered monthly water quality data were used to calibrate and validate the SWAT model. The measured and simulated streamflow values were consistent in the calibration period (2003–2010) and in the validation period (2011–2014) (Figure 4). The values of R^2^ and E_NS_ were greater than 0.5, which implied that the SWAT model’s performance was “good” for the nine hydrological stations of the study area (Table 2). The measured and simulated nutrient yield showed a good agreement in the calibration period, during 2003–2004, and the validation period, 2005 (Figure 5). The E_NS_ and R^2^ values were 0.39 and 0.36, respectively for the calibration periods at the Shenyang and Xingjiawopeng monitoring stations, which indicates a “fair” model performance for TN. The values of E_NS_ and R^2^ were greater than 0.5 at the four monitoring stations, which suggested that the model performance was “good” in simulating TP yield.

The model showed better results in simulating hydrological processes than in simulating TN and TP yields, which may be because the runoff parameters were adjusted first, nutrients were subsequently adjusted, and errors induced in adjusting parameters could be transferred and extended to the simulation [43]. Overall, the consistency between the results of the simulation and the measured values, as well as the high E_NS_ and R^2^ values, indicated that the calibrated and validated model could successfully describe monthly streamflow and nutrient yield in the study area.

**Figure 6:**
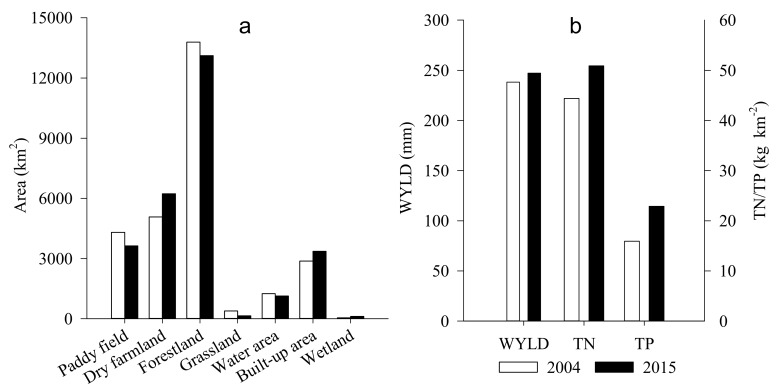
Land use area, water yield (WYLD), total nitrogen (TN) and total phosphorus (TP) yield in 2004 and 2015. (**a**) Land use area in 2004 and 2015; (**b**) WYLD and nutrient yield in 2004 and 2015.

The simulated annual WYLD and nutrient yield with the validated SWAT model showed an increasing trend from 2004 to 2015 (Figure 6). The WYLD for 2004 and 2015 was 238.15 and 247.13 mm, respectively, increasing by 8.98 mm. The TN yield increased from 44.39 kg km^−2^ in 2004 to 50.88 kg km^−2^ in 2015. The TP yield was 22.88 kg km^−2^ in 2015, with an increase of 6.97 kg km^−2^ in comparison to that in 2004.

### 3.2. Contribution of Land Use Changes to WYLD and Nutrient Yield Changes

**Figure 7:**
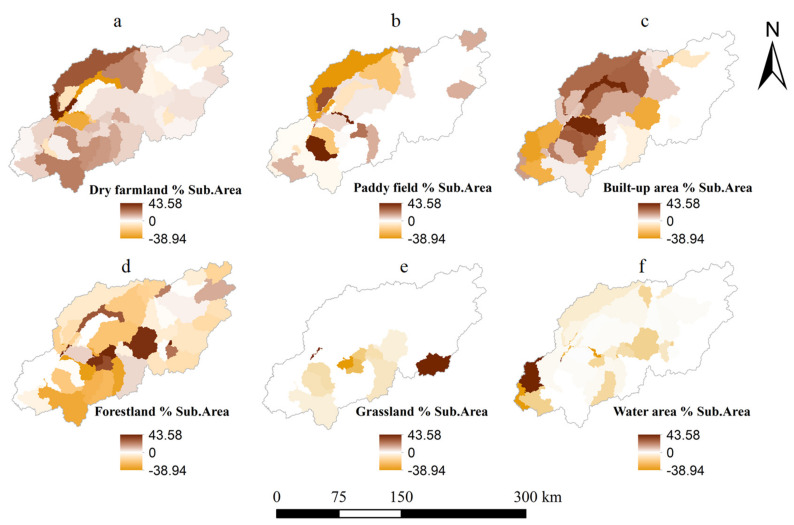
The spatial distribution of the land use changes in 2004 and 2015. The percentage of individual land use changes area in each subbasin was divided by the corresponding area of the subbasin. (**a**) The percentage of dry farmland change area ; (**b**) The percentage of paddy field change area; (**c**) The percentage of built-up area change area; (**d**) The percentage of forestland change area; (**e**) The percentage of grassland change area; (**f**) The percentage of water area change area.

**Figure 8:**
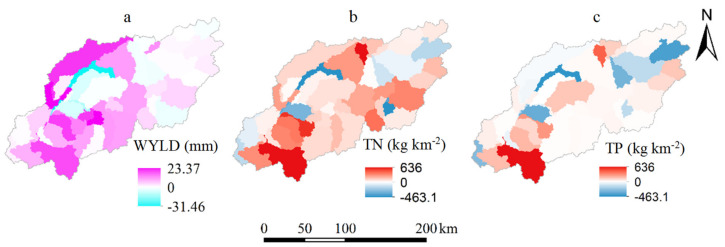
The spatial distribution of the water yield (WYLD), total nitrogen (TN) and total phosphorus (TP) yield changes in 2004–2015 based on the “fixing-changing” method. (**a**) WYLD changes in 2004–2015; (**b**) TN changes in 2004 and2015; (**c**) TP changes in 2004 and 2015.

Forestland, dry farmland, paddy field, and built-up area were the main land use categories (Figure 6), and forestland accounted for as much as 45% of the total study area. From 2004 to 2015, forestland, paddy field, grassland, and water area decreased by 56.38, 56.25, 20.30, and 9.72 km^2^ per year, respectively. Conversely, dry farmland, built-up area, and wetland increased with an annual growth by 96.06, 40.57, and 6.10 km^2^, respectively. Wetland was not considered in further analyses due to their area being less than 1% of the basin. The spatial distribution of land use changes mainly occurred in the midstream and downstream areas and were mainly in the farmland and built-up area (Figure 7, Table S1 and Figure S1). The dramatic increase in WYLD and nutrient yield also primarily occurred in the midstream and downstream areas, strongly matching the spatial distribution changes in the dry farmland and built-up area (Figure 7 and Figure 8). The decrease in WYLD and nutrient yield in the midstream area of the catchment spatially is due to the decrease of dry farmland.

Three BRT models were established to quantify the relative contribution of land use changes to WYLD, TN, and TP (Table 3). The WYLD, dry farmland, built-up area, paddy field, and water area were correlated with WYLD, and the relative contributions were 42.03%, 23.79%, 17.06%, and 13.55%, respectively. The WYLD was positively correlated with dry farmland and built-up area but negatively correlated with paddy field and water area. The changes in dry farmland (54.30%) was the most important land use related to TN, followed by forestland (22.26%), built-up area (11.50%), and water area (9.21%). The relative contribution of land use changes to TP changes in a descending order was forestland (29.78%), dry farmland (25.55%), built-up area (24.95%), and water area (15.37%). TN and TP yields were positively correlated with dry farmland, built-up area, and water area but negatively correlated with forestland. The impacts of grasslands were ignored due to their low area percentage (1.41% in 2004 and 0.53% in 2015).

### 3.3. Relationship Between Landscape Pattern Changes and WYLD and NPS changes

Prior to the RDA, the landscape indexes were selected based on values of significance and importance (Table 4). The results were inconsistent with those of the BRT model, and in comparison to that of the other land uses, the percentage of forestland and dry farmland had a greater impact on TN and TP yields, with contributions of 28% and 19.7%, respectively. The PAFRAC, AI, LSI, and LPI showed significant power in explaining WYLD and NPS loading (*p <* 0.05). The selected variables explained 52.28% of the variation in WYLD and nutrient yield. The first two RDA ordination axes explained more than 90% of the total correlation among the landscape and WYLD and nutrient yield (*p =* 0.01).

**Figure 9:**
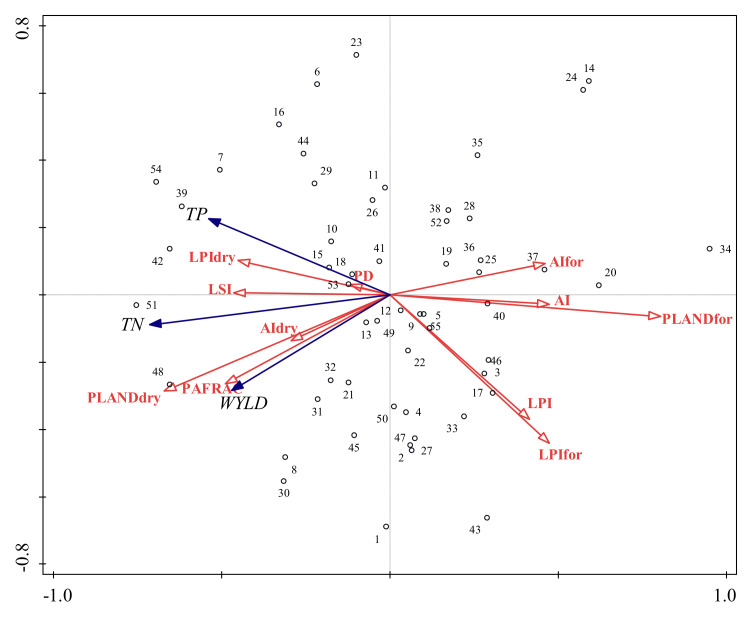
Redundancy analysis (RDA) triplots displaying the relationship among water yield (WYLD), total nitrogen (TN) and total phosphorus (TP) loading, and landscape metrics. Percentage of landscape (PLAND) of forestland (PLANDfor), PLAND of dry farmland (PLANDdry), Perimeter area fractal dimension (PAFRAC), Patch density (PD), Landscape shape index (LSI), Largest patch index (LPI), LPI of forestland (LPIfor), LPI of dry farmland (LPIdry), Aggregation index (AI), AI of forestland (AIfor), AI of dry farmland (AIdry).

The percentage of landscape (PLAND) of forestland (PLANDfor), PLAND of dry farmland (PLANDdry), Perimeter area fractal dimension (PAFRAC), Landscape shape index (LSI), 2Largest patch index (LPI), LPI of forestland (LPIfor), LPI of dry farmland (LPIdry), Aggregation index (AI), AI of forestland (AIfor), AI of dry farmland (AIdry).At the class level, the correlations between changes in WYLD and nutrient yield as well as changes in largest patch index (LPI) of dry farmland (LPIdry), aggregation index (AI) of dry farmland (AIdry) and the percentage of landscape (PLAND) of dry farmland (PLANDdry)were positive; otherwise, they were negative for LPI of forestland (LPIfor), AI of forestland (AIfor) and PLAND of forestland (PLANDfor) (Figure 9, Supplementary Materials, Figures S2 and S3). At the landscape level, WYLD and NPS loading were positively related to LSI and PAFRAC and negatively related to LPI and AI. We also found that there was a positive correlation between WYLD and nutrient yield, which demonstrated that WYLD is relatively more likely to lead to nutrient loss.

## 4. Discussion

### 4.1. Influence of Land Use Changes on WYLD and Nutrient Yield Changes

Our results showed that forestland was the dominant land used in the Hun-Taizi River watershed, which was mainly distributed in the mountain hills, and it was negatively correlated with WYLD and TN and TP yields. These results are consistent with the results of many previous studies [41,43,44]. The forestland reduces the annual streamflow in northeastern China [45,46]. Various characteristics of the forestland could have effects on WYLD, including canopy cover, litter-humus layer, root system, and evapotranspiration rate [1]. Forestland can reduce surface runoff and increase groundwater due to their capacity to intercept rainfall and increase infiltration [47]. According to our calculation, surface runoff accounts for approximately 65% of the WYLD in our study area. Hence, WYLD is reduced owing to the reduction in surface runoff. Forestland can reduce soil erosion and absorb pollutants, thus reducing pollutants from runoff into water and improving water quality.

Dry farmland was positively correlated with TN and TP yields because conventional agricultural activity (tillage or excessive fertilization) increases the risk of soil erosion and excessive nutrient applications [48]. The increase in built-up area associated with urbanization led to an increase in impervious surfaces and surface runoff [10]. In addition to surface flow, pollutants are easily washed to receiving waters, resulting in the deterioration of water quality. Nutrient yield responded positively to dry farmland, built-up area, and water area but negatively to forestland.

The results revealed that the larger the water area, the more severe the NPS pollution was. This finding differs from those of previous studies that concluded that a larger water area would have higher water quality [19,49]. This may be due to our study area being located in the central Liaoning Urban Agglomeration with a high level of urbanization, industrialization, and pollution. In addition, the Hun River and Taizi River are rain-fed rivers, and precipitation is mainly concentrated in summer. An increase in impervious surfaces will wash away more contaminants. Even small rain events are capable of washing pollutants from impervious surfaces into receiving waters. The amount of pollutants entering water can exceed its dilution ability if that amount increases. The results indicated that NPS pollution became the main reason for water quality degradation.

The paddy field could reduce WYLD with a relative contribution of 23.42%; however, this reduction was not significant in terms of nutrient yield. NPS loading from the paddy field largely depends on management practices and precipitation [50]. Farmers always keep the paddy field flooded until rice is harvested. When the rainfall is minimal, there is no surface runoff and less infiltration [19]. However, heavy rain will lead to the fertilized paddy field overflow into adjacent waters, which will have a negative impact on water quality. In our study periods (2004 and 2005), there was no abnormally high precipitation in the watershed; therefore, the paddy field acted as a pool for precipitation. Therefore, an increase in the paddy field can reduce WYLD, and the relationship with NPS pollution is not significant.

### 4.2. Influence of Landscape Pattern Changes on WYLD and NPS changes

At the class level, WYLD and nutrient yield were negatively correlated with the LPI and AI of forestland and positively correlated with the LPI and AI of dry farmland (Figure 9), which is consistent with results from related studies [4,41,51]. The annual evapotranspiration (ET) losses of crops are generally smaller than the ET losses from trees [51,52]. The change from forestland to farmland resulted in decreased ET, thereby increasing WYLD [34]. Preferred forestland patterns to improve water quality appear to be those that are concentrated and have a high value for the largest patch proportion, which can effectively reduce surface runoff and intercept nutrient elements [53]. Dry farmland with a larger patch area will lead to an increase in nutrient yield because dry farmland is the main source of NPS pollution. Thus, large unfragmented forestland patches may improve water quality, and large, concentrated patches of dry farmland may adversely affect water quality.

At the landscape level, our findings showed that WYLD and nutrient yield were positively correlated with LSI and PAFRAC but negatively correlated with LPI and AI [21,44,54]. PD was positively correlated with LSI and PAFRAC while negatively correlated with LPI and AI (Figure 9), which implies that the values of LSI and PAFRAC increased and those of LPI and AI decreased, and the landscape will be more fragmented and highly influenced by intense human activities. Due to the intense human activities the area of the impervious surface increased, which decreased the evapotranspiration and the infiltration of precipitation, consequently increasing WYLD [55]. Small areas of various land uses negatively affect water quality [4]. If the shape of the landscape complex, the patch area is small and the patches are scattered, which are unfavorable for inhibiting soil erosion and runoff, then pollution will be more likely to runoff into waterbodies [44]. Our results suggest that in a forestland-dominated watershed, water quality is more likely degraded when land uses are scattered with many small patches.

### 4.3. Implications

Forestland is the dominant land use in the study area, which occupies the mountain areas in the upper watershed, and an increase in the proportion of unfragmented forestland with a large patch area was positively related to water quality. Therefore, increasing the area and concentration of the forestland is of great significance for water conservation and control of NPS pollution. According to the results of this study, dry farmland is the major source of NPS pollution, and the AI and LPI of dry farmland had a strong negative impact on water quality. Therefore, encouraging farmers to rationally use chemical fertilizers and pesticides, as well as conservation tillage would help to reduce nutrient loss. Landscape planning, such as increasing patch aggregation and decreasing landscape fragmentation, should be considered to improve watershed water quality.

The SWAT model was widely used to study the influence of land use or climate changes to the streamflow, sediment, or TN and TP yields. To ensure the accuracy of the SWAT model, it was calibrated and validated under the real condition while the model was used to simulate the future hydrology or NPS loading under different land use scenarios or future climate scenarios [5,56,57]. In some research, the SWAT model was calibrated and validated just in a short period or even not validated [16,19,58,59] due to the data that are not readily available in China [39]. Once the calibration and validation was completed, the same input parameters were used with a different set of data, such as different climatological and land-use conditions. In our study, calibration and validation of TN and TP was acceptable for only three years, which may bring uncertainties of the SWAT.

With population growth, economic development, and urban sprawl, the built-up area greatly increased in the Hun-Taizi River watershed [60]. A large amount of farmland was occupied by the built-up area, inducing a contradiction between the limited arable land resources and the increasing rural labor. In this situation, an increasing number of rural migrants led to a decrease in intensive cultivation activities. The “Red Line of 1.8 Billion Mu of Agricultural Land” policy was proposed in 2006, forming stricter farmland protection. Given agricultural activities and urbanization, the sources of NPS pollutants have become more complex, leading to a serious NPS pollution in highly urbanized basins, especially in urban agglomeration watersheds. Most urban agglomerations in China are located in the same river basin and face similar issues. How to address the relationship among rapid urbanization, stable farmland, and NPS pollution is a great challenge for water quality conservation in China.

The built-up area has a great influence on water quality at smaller scales [21,61]. However, in our study, the contribution of the built-up area to TN only accounted for 5.85%. On the one hand, the SCS curve number used by the SWAT to calculate the surface runoff considered the built-up area as an impervious surface and ignored spatial patterns such as pipe networks, leading to an underestimation of the urban NPS loading amount. On the other hand, transport and fate processes in-stream were usually ignored or simplified and described using QUAL2E equations, which may affect the SWAT modeling accuracy [62]. Therefore, future research on highly urbanized watersheds should focus on the accurate estimation of urban and agricultural NPS pollution by integrating models. A coupling analysis of the BRT and RDA methods in this paper was useful and novel and quantitatively analyzed the relative contribution/correlation of land use/landscape pattern changes to WYLD and NPS pollution.

## 5. Conclusions

The SWAT model performance was “good” and “fair” for simulating streamflow with TP (R^2^ and E_NS_ were greater than 0.5) and TN, respectively. The SWAT model performed better in simulating the hydrological process than in simulating nutrient yield due to the accumulated parameter errors (runoff parameters adjusted first and then nutrient parameters).

WYLD was strongly correlated with dry farmland, built-up area, paddy field, and water area, with contributions of 42.03%, 23.79%, 17.06%, and 13.55%, respectively. Forestland was the dominant land use in the catchment and was negatively correlated with WYLD and NPS loading due to its ability to reduce surface runoff and soil erosion and absorb pollutants. Human activities were the main cause of NPS pollution. Dry farmland and built-up area were the main sources of NPS pollution, while paddy field had no significant effect on nutrient yield because they were always flooded and acted as a pool. The water area was positively correlated with NPS loading, indicating that NPS pollution was the main reason for water quality degradation.

Landscape patterns had an important influence on WYLD and NPS pollution. Large unfragmented forestland may improve water quality, while large concentrated dry farmland may adversely affect water quality. NPS pollution will be severe when various land uses have a complex shape and are dispersed and fragmented in a forestland-dominated watershed. Forestland plays an important role in controlling NPS pollution. The results of this study can help resource managers and government policy makers develop scientific and effective strategies for land use and water resource management. The BRT and RDA methods are simple and feasible and can be used in other basins of China.

## Figures and Tables

**Table 1:** Soil and Water Assessment Tool (SWAT) model parameters.

Data	Data Format	Data Description	Data Source
Digital elevation model (DEM)	Vector map (1:5000)	Elevation, slopes (Figure 2a)	Liaoning Surveying and Mapping Bureau
Soil map and properties	Grid (cell size, 0.0083333333°)	Physical and chemical properties of soils (Figure 2b)	Harmonized World Soil Database (Version1.2) (http://www.fao.org/nr/land/soils/harmonized-world-soil-database/en/)
Land use map	Grid (cell size, 30 × 30 m)	Land use classification in 2004 (Figure 2c) and 2015 (Figure 2d)	Landsat 5 Thematic Mapper and Landsat 8 Operational Land Imager (https://earthexplorer.usgs.gov/)
Weather	Database file (DBF)	Including daily data of precipitation, maximum and minimum temperature, humidity and wind speed	China Meteorological Administration (https://data.cma.cn/); Local Bureau of Meteorology
Hydrology and water quality data	Database file (DBF)	Monthly observed streamflow data, maximum and minimum discharge data of the four reservoirs, and monthly observed nutrient yield	Local hydrographical station and environmental monitoring station
Agricultural management	Database file (DBF)	Including crop planting time, fertilization, and harvested time	Liaoning statistical bureau and field survey
Point and nonpoint source data	Database file (DBF)	PS: Urban population and industrial production NPS: Rural population and livestock rearing	Liaoning statistical yearbooks (http://www.ln.stats.gov.cn/tjsj/sjcx/ndsj/)

PS—Point source; NPS—Nonpoint source.

**Table 2:** The E_NS_ and R^2^ values for the Soil and Water Assessment Tool (SWAT) calibration and validation. ”-” means missing observed data in validation period.

	Stations	Calibration		Validation	
		R^2^	E_NS_	R^2^	E_NS_
Streamflow	Beikouqian	0.80	0.77	0.88	0.87
	Fushun	0.95	0.90	0.99	0.98
	Shenyang	0.86	0.77	0.80	0.78
	Huanglatuo	0.85	0.80	0.82	0.80
	Xingjiawopeng	0.82	0.77	0.78	0.78
	Benxi	0.72	0.63	0.68	0.65
	Laioyang	0.64	0.58	0.67	0.64
	Xiaolinzi	0.70	0.64	0.72	0.69
	Tangmazhai	0.73	0.66	0.76	0.74
TN	Shenyang	0.63	0.39	0.95	0.87
	Xingjiawopeng	0.76	0.36	0.78	0.58
	Liaoyang	0.94	0.78	-	-
	Xiaolinzi	0.91	0.73	-	-
TP	Shenyang	0.92	0.66	0.91	0.61
	Xingjiawopeng	0.77	0.55	0.90	0.81
	Liaoyang	0.66	0.65	0.90	0.72
	Xiaolinzi	0.96	0.90	0.66	0.58

**Table 3:** Summary of the relative contributions of land use changes in the boosted regression tree (BRT) models for water yield (WYLD), total nitrogen (TN), and total phosphorus (TP).

	WYLD	TN	TP
Dry farmland	+42.03	+54.30	+25.55
Paddy field	−17.06	−2.72	−4.35
Forestland	−3.58	−22.26	−29.78
Grassland	0	0	0
Water area	−13.55	+9.21	+15.37
Built-up area	+23.79	+11.50	+24.95

“+” or “-” represent positive or negative correlations of WYLD and nutrient yield changes with land use changes, respectively, which were determined from the partial dependency plot.

**Table 4:** Selected landscape indexes for further redundancy analysis (RDA) by the Monte Carlo permutation test (*n* = 499).

Variables	Contribution (%)	Significance	Variables	Contribution (%)	Significance
PLANDfor	28.0	0.002	LSI	9.3	0.02
PLANDdry	19.7	0.006	AIfor	9.2	0.034
PAFRAC	10.5	0.008	LPIdry	8.9	0.036
LPIfor	10.2	0.012	AIdry	8.5	0.038
AI	9.7	0.028	LPI	7.8	0.04

## Supplementary Materials

The following are available online at https://www.mdpi.com/1660-4601/17/9/3060/s1; Table S1: The land use change areas in the Hun-Taizi River watershed from 2004 to 2015 (units, km^2^). Figure S1: The main land use change map in the Hun-Taizi River watershed from 2004 to 2015. Figure S2: RDA analysis triplots displaying the relationship among WYLD, NPS loading, and landscape metrics in high hills. Figure S3: RDA analysis triplots displaying the relationship among WYLD, NPS loading, and landscape metrics in plain.
