# Supplementary material for: Effects of Landscape Pattern Change on Water Yield and Nonpoint Source Pollution in the Hun-Taizi River Watershed, China
Source: Int J Environ Res Public Health. 2020 Apr 28;17(9):3060. doi: 10.3390/ijerph17093060 (PMC7246484; doi:10.3390/ijerph17093060)
Supplement: Supplementary file 1 [file ijerph-17-03060-s001.pdf]

International Journal of
Environmental Research and Public Health

Supplementary Material

Table S1. The land use change areas in the Hun-Taizi River watershed from 2004 to 2015（units, km
）

	Paddy field	Dry farmland	Forestland	Grassland	Water area	Built-up area	Wetland
Paddy field	2792.15	926.06	67.04	5.96	13.78	462.68	41.59
Dry farmland	477.50	3406.70	598.82	22.44	0.01	559.13	10.34
Forestland	98.40	1349.53	12031.80	98.32	0.15	203.95	7.58
Grassland	5.69	93.71	252.23	10.49	0	27.66	0.12
Water area	33.98	27.21	27.40	1.12	1118.90	25.40	16.66
Built-up area	225.80	422.02	134.16	5.52	1.16	2085.40	4.14
Wetland	0.77	2.43	0.78	2.52	0	0.79	38.57

The "row" represents 2004 land use, and the "column" represents 2015 land use.

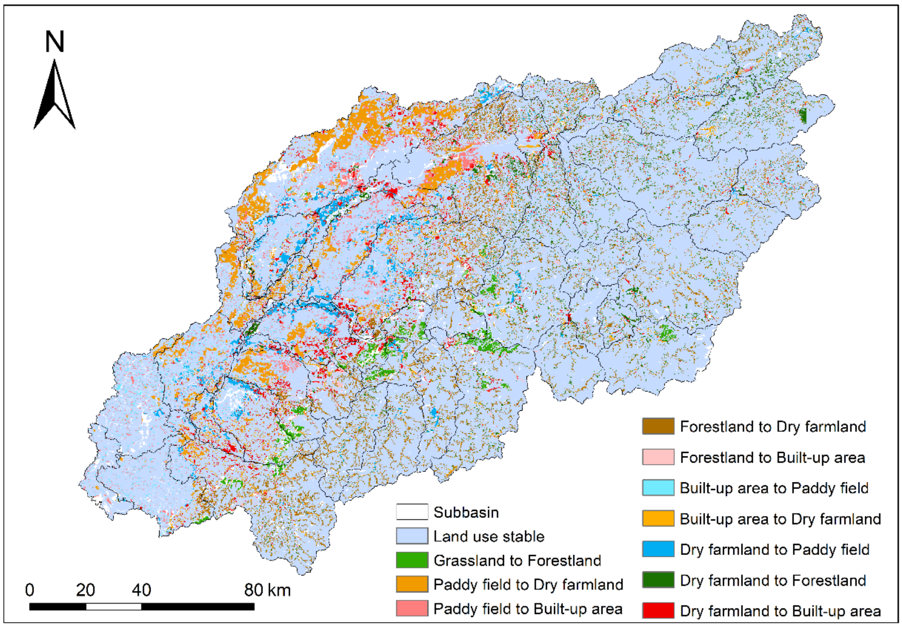

Figure S1. The main land use change map in the Hun-Taizi River watershed from 2004 to 2015.

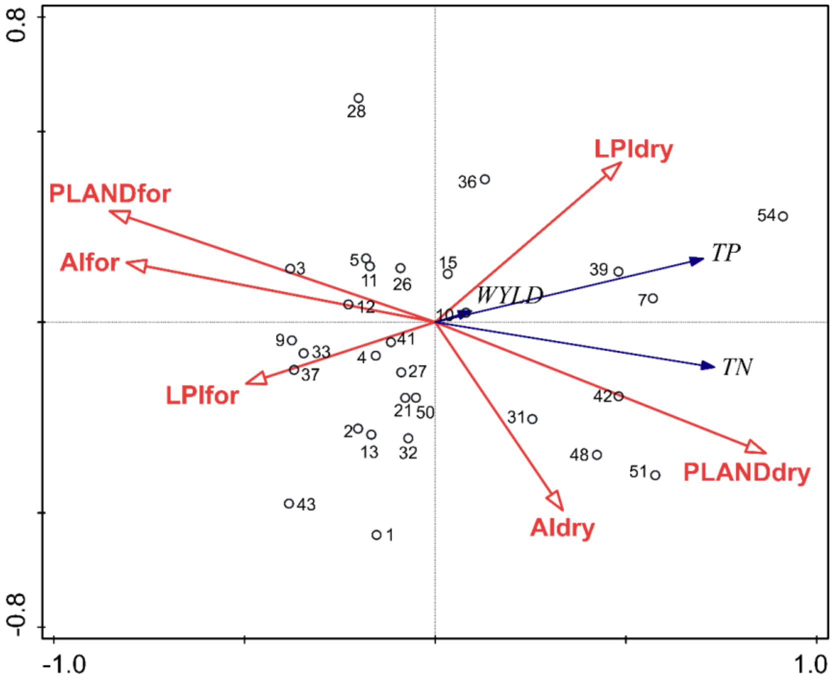

Figure S2. RDA analysis triplots displaying the relationship among WYLD, NPS loading, and landscape metrics in high hills.

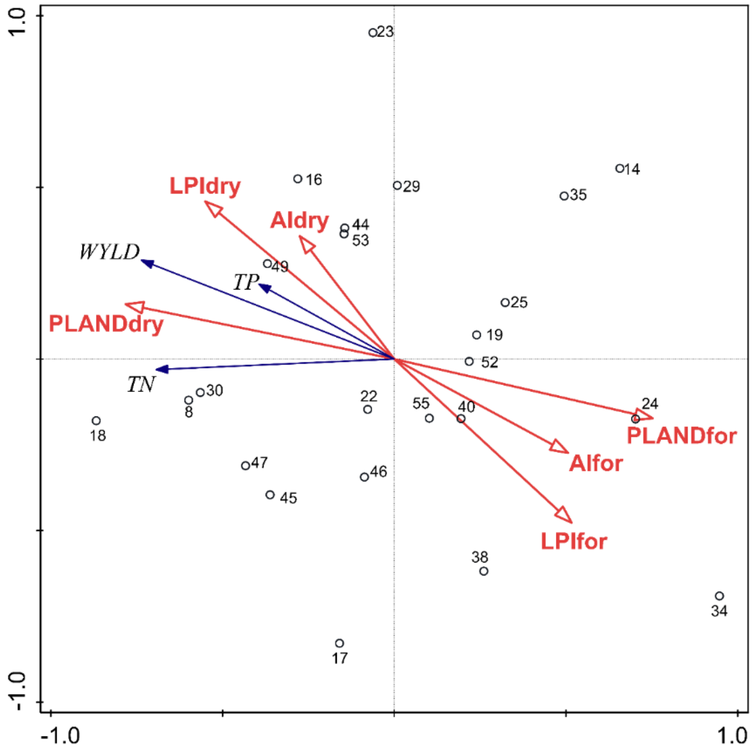

Figure S3. RDA analysis triplots displaying the relationship among WYLD, NPS loading, and landscape metrics in plain.
